# A randomised clinical trial comparing a surgical approach for treatment of peri-implantitis to non-surgical debridement with adjunctive diode laser therapy

**DOI:** 10.1007/s00784-025-06204-9

**Published:** 2025-02-19

**Authors:** Dena Hashim, Delphine Courvoisier, Norbert Cionca

**Affiliations:** 1 Division of Regenerative Dental Medicine and Periodontology, University Clinics of Dental Medicine, Faculty of Medicine, University of Geneva, 1 Rue Michel-Servet, CH-1211 Geneva 4, Switzerland; 2 University Hospitals of Geneva HUG, Rue Gabrielle-Perret-Gentil 4, CH-1205 Geneva, Switzerland

**Keywords:** Peri-implantitis, Laser therapy, Diode laser, Therapeutics, Surgery, Treatment outcome, Dental implants, Clinical trial

## Abstract

**Objectives:**

To evaluate the efficacy of non-surgical debridement with repeated diode laser application in comparison to surgical treatment for management of peri-implantitis.

**Materials and methods:**

Forty patients diagnosed with peri-implantitis were randomised into two groups. The test group received mechanical debridement and repeated diode laser therapy at Days 0, 7 and 14. The control group received mechanical debridement at Day 0 followed by surgical treatment at Day 14. Clinical evaluations were performed at baseline, 3 and 12 months.

**Results:**

Thirty-six participants (test *n* = 17, control *n* = 19) completed the 12-month observation period. Laser treatment failed in 4 cases (23.5%); of which 3 implants lost osseointegration and one necessitated surgical treatment due to progressively increasing probing depths (PD) and bone loss. In comparison, the control group showed a 100% survival rate with a statistically significant difference between the two groups (*p* = 0.04). Therefore, thirty-two participants were examined at the final evaluation (test *n* = 13, control *n* = 19). Twenty-two implants (57.9%) showed complete disease resolution without significant differences between the groups. The test group reported significantly lower post-operative discomfort on the visual analogue scale (VAS). At 3 months, both groups showed clinical signs of healing with reduction in probing depths (PD) and bleeding upon probing. Surgical treatment resulted in significantly lower PDs (control 3.7 mm [3.2, 4.0], test 4.5 mm [3.8, 4.8]), but recession was significantly higher (control 0.5 mm [0.3, 1.2], test 0 mm [0.0, 0.3]). At the final reevaluation, PD values remained significantly lower in the control group; 3.3 mm [3.1, 3.9] compared to 4.3 mm [3.7, 4.8] for the test group, but the difference in mucosal recession fell below the level of significance. Marginal bone levels improved after one year without significant differences between the two groups (Test = 3.5 mm [2.8, 4.6] at baseline and 1.5 mm [1.0, 4.4] at one year, Control = 2.8 mm [2.5, 3.1] at baseline and 1.4 mm [1.0, 2.6] at one year).

**Conclusion:**

Surgical approaches for management of peri-implantitis demonstrated significant benefits over laser therapy in terms of treatment success and PD reduction. Nevertheless, diode laser therapy, as described in this study, could represent a minimally invasive alternative for treatment of non-advanced peri-implantitis defects.

## Introduction

Peri-implantitis is a common inflammatory disease affecting functional oral implants. It is associated with pathological bone loss and pocket formation [12]. Progressive peri-implantitis may lead to both aesthetic and functional complications, and eventually even implant loss [50]. Numerous studies have predicted escalation of peri-implantitis in the upcoming future, with an already estimated prevalence of 56% and an increasing incidence from 0.4% to 43.9% within 3 to 5 years [24, 43, 70]. Therefore, different treatment approaches have been extensively evaluated over the years. This encompassed a wide variety of clinical protocols that generally include mechanical debridement, implant surface decontamination and resective or bone regenerative measures [13, 16, 17, 19, 21, 22, 35, 40]. Still, studies have failed to identify an ideal treatment approach leading to consistent and complete resolution of the inflammatory lesions [27, 33, 37, 54, 57, 69]. Success rates greatly varied from one trial to the other (25–45% at 12 months), and no particular protocol could be proven predictably effective in controlling the disease on the long term [2]. The scarcity of high quality scientific evidence and the absence of a true control group is a common limitation [29]. Nonetheless, consensus statements generally recommend mechanical debridement, followed by early evaluation, then surgical treatment in case of unresolved peri-implantitis lesions. [31, 37].

When an implant is affected by peri-implantitis, its exposed rough surface is coated by a biofilm of mixed anaerobic bacteria [8, 9, 23]. The titanium’s surface roughness, as well as its high wettability and surface energy, further increase bacterial adhesion and colonisation. Therefore, elimination of the bacterial biofilm is an absolute prerequisite for treatment of peri-implantitis [58, 60]. Decontamination techniques should ideally produce a biocompatible surface which promotes re-osseointegration of the exposed portion of the implant [3, 46, 63]. Numerous techniques have been tested over the years, ranging from a variety of antiseptic solutions, photodynamic therapy, hand or ultra-sonic instrumentation, air-powder abrasives, and different types of lasers. Yet the clinical superiority of one method over the other is still undetermined [27, 30, 64]. Nevertheless, laser therapy has been recently attracting significant attention [5, 7, 42, 52]. Diode lasers in particular are known for their bactericidal, photobiomodulatory and tissue ablation effects, which have made them increasingly popular for peri-implantitis treatment [5, 41, 66, 67]. *In vitro* experiments had further highlighted diode laser’s ability to inhibit lipopolysaccharide-induced macrophage activation, reduce inflammation and promote osseointegration and tissue regeneration [28, 67]. However, research has thus far been limited to animal studies, *in vitro* investigations and clinical trials with small samples and short follow-up periods [6, 7, 38, 41, 47, 66].

Laser treatment offers a non-invasive decontamination method and promotes healing of the peri-implant inflammatory lesion. Its bactericidal effects may further eliminate the need for systemic antimicrobials traditionally required following conventional surgical treatment, thereby reducing their eminent threat to human health in general [11]. Therefore, this randomised controlled trial aims to evaluate the clinical and patient-centred outcomes of non-surgical mechanical debridement with repeated diode laser therapy in comparison to surgical treatment for management of peri-implantitis.

## Materials and methods

This is a prospective, single-center, superiority, examiner-blinded, randomised, controlled clinical trial of 12 months duration. The study was conducted in compliance with the current version of the Declaration of Helsinki, the ICH-GCP or ISO EN 14155 as well as all national legal and regulatory requirements. The protocol was developed according to the CONSORT guidelines for clinical trials and was registered at ClinicalTrials.gov (ID no. NCT03383120).

### Patient selection and randomisation

Participants were selected from the pool of patients being treated at the University of Geneva Clinics of Dental Medicine, Geneva, Switzerland. Patients with at least one screw-type titanium implant diagnosed with peri-implantitis were included in this study. Peri-implantitis was defined as at least one site with probing depth (PD) ≥ 5 mm, bleeding upon probing (BOP) and/or suppuration (SUP), and radiographic evidence of marginal bone loss (MBL) ≥ 2 mm after at least one year of insertion of the final prosthetic restoration. When more than one implant fit the criteria, the one with the most advanced marginal bone loss was selected.

Exclusion criteria included systemic conditions that could compromise wound healing; such as uncontrolled diabetes mellitus, cancer or bone metabolic disorders, radiation or immunosuppressive therapy. Patients with known hypersensitivity to penicillin and/or metronidazole, women who are pregnant or lactating, known or suspected non-compliance, drug or alcohol abuse, heavy smokers consuming > 10 cigarettes/day and untreated periodontal disease, were also excluded.

Forty patients (22 males and 18 females) were enrolled in this study between October 2017 and November 2018 with signed informed consent. The sample size was calculated based on previous studies performed at the time of the protocol’s development  [33, 65]. Participants were randomly distributed by a computer-generated table to one of two parallel groups; the test group (*n* = 20) receiving mechanical debridement and adjunctive repeated diode laser therapy, and the control group (*n* = 20) receiving initial mechanical debridement followed by surgical treatment. The random allocation sequence was password-protected and only accessible to the operator performing all interventions. The patients were not blinded to the treatment group but were instructed to avoid disclosing their group allocation to the blinded examiner throughout the study period.

### Baseline examination

Clinical examination was conducted by one experienced examiner (NC) blinded to the treatment group. Baseline measurements of affected implants included PD, mucosal recession (REC), BOP and SUP. The presence or absence of ≥ 2 mm of keratinised gingival tissue (KT) was also recorded. A periodontal probe (12 UNC colour-coded) was used to evaluate PD, BOP and REC at 6 sites per implant. The presence of plaque was also recorded at 6 sites while SUP was only noted as present or absent on an implant level. Standardised intra-oral radiographs were taken using a long-cone paralleling technique with a RINN holder, and full-mouth plaque scores (PS) were recorded. Baseline MBL was assessed by two independent examiners (DH, NC) at the mesial and distal aspects of each implant, and mean values were calculated for each examiner. The mean inter-examiner MBL values (mMBL) were utilised for analysis.

### Treatment

Prior to treatment, all participants were enrolled in an oral hygiene program including professional supra-gingival scaling and polishing as well as oral hygiene instructions. One experienced operator (DH) performed all active treatment procedures. Under local anaesthesia, all participants received sub-gingival mechanical debridement using an ultrasonic device (EMS Piezon®) with a special tip and sub-mucosal saline irrigation. The control group did not receive any further treatment during this visit.

For the test group, curettes were used to remove sub-gingival granulation tissue and pocket epithelium prior to sub-mucosal diode laser therapy (settings: 810 nm, 2.5 W, 50 Hz, 10 ms). The implants were irradiated 3 times for 30 s, using a 400-µm thick fibre according to the manufacturer’s instructions (Wiser diode laser®, Orcos Medical AG, Küsnacht, Switzerland). The laser tip was moved both vertically and horizontally in a scanning manner along the contaminated implant surface. To prevent hotspots, the tip was checked every 4–5 s for coagulation. In case of a hotspot, the fibre tip was cut-off with scissors to avoid heat development. Trans-gingival laser biostimulation with a low-level diode laser was finally performed for 60 s (settings: 810 nm, 1 W/cm^2^) in circular movements. This was repeated 7 and 14 days later as previously described by Mettraux et al. [47].

The control group received surgical treatment 14 days following initial sub-gingival debridement. Under local anaesthesia, open flap debridement and implant surface decontamination using a 0.9% normal saline solution were performed. In the presence of deep well-contained intra-osseous defects, guided bone regeneration (GBR) was performed using xenogeneic particulate bone grafts and resorbable collagen membranes (Bio-Oss® and Bio-Gide® respectively, Geistlich Pharma AG, Switzerland). GBR was deemed necessary for three- or two-wall osseous defects with at least 3 mm infra-bony depth. Implantoplasty was performed in cases with horizontal bone loss and exposed implant spears, depending on accessibility and only for posterior non aesthetic cases. All control subjects received post-operative systemic antibiotics (Amoxicillin 500 mg 3x/day and Metronidazole 500 mg 3x/day for one week) along with a Chlorhexidine mouth rinse 2x/day and non-steroidal anti-inflammatory drugs.

For all participants, adverse events were recorded and managed when necessary. Patients’ level of post-operative discomfort were assessed using a visual analogue scale (VAS) at each treatment visit.

### Follow-up

The first follow-up visit was scheduled 7–10 days following termination of the active treatment phase. Adverse events were recorded, and patients’ level of discomfort was assessed using the VAS. Sutures were removed and oral prophylaxis was performed for the control group. The operator (DH) oversaw this visit to maintain the blinding of examiners. Additional follow-ups were planned one and two months post-operatively. Oral prophylaxis was performed, and hygiene instructions were reinforced when required.

### Reevaluation and maintenance

Three months after completion of the therapeutic phase, the examiner (NC) performed the first reevaluation visit. Clinical measurements were recorded as previously described during the baseline examination. In case of increasing PD with concomitant BOP and/or suppuration, the patients were referred back to the operator (DH) for rescue treatment. The test group received submucosal ultrasonic debridement and adjunctive diode laser therapy for 90 s, as previously described. The control group received submucosal ultrasonic debridement only. All patients were then enrolled in a maintenance program 6 and 9 months post-operatively. The final reevaluation was performed 12 months following completion of the active treatment, i.e., surgical treatment for the control group and the third laser visit for the test group. Again, clinical and standardised radiographic examinations were performed by the initial examiner (NC). The examiner was blinded to the treatment group at all time points. Participants’ overall perception of the treatment was also evaluated using VAS scores at the end of the observation period.

### Outcomes

The primary outcome measure was the resolution of the peri-implantitis lesion. This was defined as absence of peri-implant sites with PD > 4 mm, BOP and/or SUP, as well as absence of progressive MBL at the end of the observation period.

Secondary outcomes included differences between the two groups regarding changes in PD and peri-implant REC levels between baseline, 3 and 12 months. Changes in marginal bone levels were evaluated at the one year visit compared to baseline. The frequency of adverse events and patients’ post-operative discomfort were also considered as secondary outcomes.

### Statistical analysis

Results are presented as medians and interquartile ranges (IQR) unless otherwise indicated. Groups were compared using Fisher exact test for categorical variables and Wilcox rank sum test for continuous variables. The main analyses were made according to intention to treat (ITT); considering all patients even those who dropped out of the study. Secondary analyses were per protocol (PP) and included only patients who were followed-up until the end of the study. For the ITT analyses, missing values due to loss of follow-up were imputed using last observation carried forward (LOCF). All tests were two-tailed with a significance threshold set at 0.05. Analyses were done by an experienced statistician (DC) using R software v.4.1.0.

## Results

Out of the 40 initially recruited participants, one failed to attend several study visits and another displayed poor compliance. Both were therefore excluded from the study. The remaining 38 participants (test group *n* = 19, control group *n* = 19) included 20 male and 18 females, with a mean age of 70.2 ± 11.3 and 67.4 ± 10.7 years for the test and control groups; respectively. Ten subjects (26.3%) were smokers; 7 (36.8%) in the test group and 3 (15.8%) in the control group. Nineteen implants (50%) were in function for 5–10 years, 11 (28.9%) for 3–5 years, and only one implant from the control group was loaded 1–3 years earlier. Thirty-two implants (84.2%) had a tissue-level design while the remaining 6 (15.8%) had bone-level configurations. Twenty-three implants (60.5%) were restored with single crowns (SC). Of these, 13 (34.2%) were cemented, 9 (23.7%) were screw-retained and one patient (2.6%) lost his crown several months prior to enrolment in this study. A single subject presented with a screw-retained cantilever restoration (2.6%) while 7 others had short-span implant-supported fixed dental prostheses (FDP, 5 (13.2%) cemented, 2 (5.3%) screw-retained). Seven patients (18.4%) had implant-supported removable dental prostheses (RDP). Table 1 details both patient and implant demographics.

**Table 1:** Demographic data describing included participants and implant characteristics

	Total (*n* = 38)	Test group (*n* = 19)	Control group (*n* = 19)
Mean age ± SD		70.2 ± 11.3 yrs	67.4 ± 10.7 yrs
Gender			
Male (%)	20 (52.6%)	11 (57.9%)	9 (47.4%)
Female (%)	18 (47.4%)	8 (42.1%)	10 (52.6%)
Smokers < 10 cig/day	10 (26.3%)	7 (36.8%)	3 (15.8%)
Implant characteristics			
Maxillary implants	25 (65.8%)	11 (57.9%)	14 (73.7%)
Mandibular implants	13 (34.2%)	8 (42.1%)	5 (26.3%)
Posterior position	30 (78.9%)	16 (84.2%)	14 (73.7%)
Anterior position	8 (21.1%)	3 (15.8%)	5 (26.3%)
Tissue-level design	32 (84.2%)	15 (78.9%)	17 (89.5%)
Bone-level design	6 (15.8%)	4 (21.1%)	2 (10.5%)
Loading duration			
1–3 yrs	1 (2.6%)	0	1 (5.3%)
3–5 yrs	11 (28.9%)	9 (47.4%)	2 (10.5%)
5–10 yrs	19 (50%)	7 (36.8%)	12 (63.2%)
> 10 yrs	6 (15.8%)	3 (15.8%)	3 (15.8%)
Unknown	1(2.6%)	0	1 (5.3%)
Prosthetic restoration			
Single crowns (SC)			
Cemented SC	13 (34.2%)	8 (42.1%)	5 (26.3%)
Screw-retained SC	9 (23.7%)	6 (31.6%)	3 (15.8%)
Lost SC	1 (2.6%)	0	1 (5.3%)
Fixed dental prosthesis (FDP)			
Cemented FDP	5 (13.2%)	0	5 (26.3%)
Screw-retained FDP	2 (5.3%)	1 (5.3%)	1 (5.3%)
Screw-retained Cantilever	1 (2.6%)	0	1 (5.3%)
Removable dental prosthesis (RDP) locator attachments	7 (18.4%)	4 (21.1%)	3 (15.8%)

**Fig. 1:**
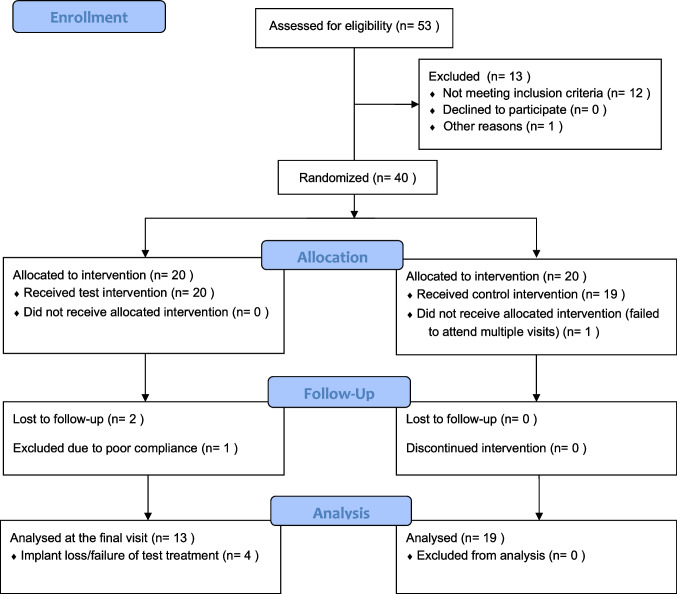
Consort flow chart

Two participants from the test group were lost during the follow-up period; one due to a severe biking accident while the other moved abroad. The ITT sample was thus composed of 38 patients and the PP sample was composed of 36 patients. Laser treatment failed in 4 out of the remaining 17 test implants (23.5%), compared to none from the control group, and the difference was statistically significant (*p* = 0.04). Failure was defined as loss of osseointegration, progressive increase in PD with repeated BOP and/or SUP, or progressive bone loss despite rescue treatment. Therefore, 32 implants were available for examination at the 12-month reevaluation visit (Fig. 1). The 4 failed implants were imputed the value of the previously available visit (LOCF). Of the 19 control subjects, nine (47.4%) received open flap debridement, 6 (31.6%) required implantoplasty, and only 4 (21.1%) underwent additional GBR treatment.

### Clinical and radiographic parameters

Baseline clinical examination did not reveal any statistically significant differences between the test and control groups regarding initial PD, BOP, SUP, REC, KT or PS (Table 2). Initial mMBL; however, was significantly more advanced in the test group compared to the control group; (test group 3.5 mm [2.8, 4.6], control group 2.8 mm [2.5, 3.1], *p* = 0.02, Table 2). A total of 92.5% of sites initially presented with BOP; 95.6% in the test group and 89.5% in the control group. Suppuration was only noted around 4 implants (21.1%); 2 from each study group. 31.6% of implants presented with at least one site < 2 mm KT; 26.3% in the test group and 36.8% in the control group. Full mouth PS was 26 ± 9 in the test group and 25 ± 10 in the control group.

**Table 2:** Results of the clinical and radiographic examinations at the baseline, 3 and 12 months reevaluation visits; presented as medians and interquartile ranges [IQR]

	Total (*n* = 38)	Test group (*n* = 19)	Control group (*n* = 19)	*P* value
Probing depth (PD) mm				
PD Baseline	5.3 [4.9, 5.7]	5.5 [5.1, 5.8]	5.3 [4.8, 5.6]	0.32
PD Reevaluation 3 m	4.0 [3.4, 4.6]	4.5 [3.8, 4.8]	3.7 [3.2, 4.0]	**0.01**
PD Reevaluation 12 m	3.8 [3.2, 4.6]	4.3 [3.7, 4.8]	3.3 [3.1, 3.9]	**0.01**
Bleeding on probing (BOP)				
BOP Baseline	1.0 [1.0, 1.0]	1.0 [1.0, 1.0]	1.0 [0.8, 1.0]	0.15
BOP Reevaluation 3 m	0.3 [0.0, 0.3]	0.3 [0.2, 0.4]	0.3 [0.0, 0.3]	0.44
BOP Reevaluation 12 m	0.3 [0.2, 0.5]	0.3 [0.2, 0.6]	0.3 [0.1, 0.3]	0.46
Recession (REC) mm				
REC Baseline	0.0 [0.0, 0.3]	0.0 [0.0, 0.2]	0.2 [0.0, 0.3]	0.39
REC Reevaluation 3 m	0.3 [0.0, 0.8]	0.0 [0.0, 0.3]	0.5 [0.3, 1.2]	**0.02**
REC Reevaluation 12 m	0.3 [0.0, 1.0]	0.0 [0.0, 0.8]	0.5 [0.2, 1.2]	0.15
Mean marginal bone loss (mMBL) mm				
mMBL Baseline	2.9 [2.5, 4.1]	3.5 [2.8, 4.6]	2.8 [2.5, 3.1]	**0.02**
mMBL Reevaluation 12 m	1.5 [0.9, 3.0]	1.5 [1.0, 4.4]	1.4 [1.0, 2.6]	0.34
VAS				
Post-operative VAS	0.0 [0.0, 3.0]	0.0 [0.0, 0.0]	3.0 [0.0, 12.0]	**0.01**
Final VAS	0.0 [0.0, 3.0]	0.0 [0.0, 3.0]	0.0 [0.0, 3.0]	0.57

*P* values represent differences between the two groups. The Fisher exact test was used for categorical variables and the Wilcox rank sum test for continuous variables

**Fig. 2:**
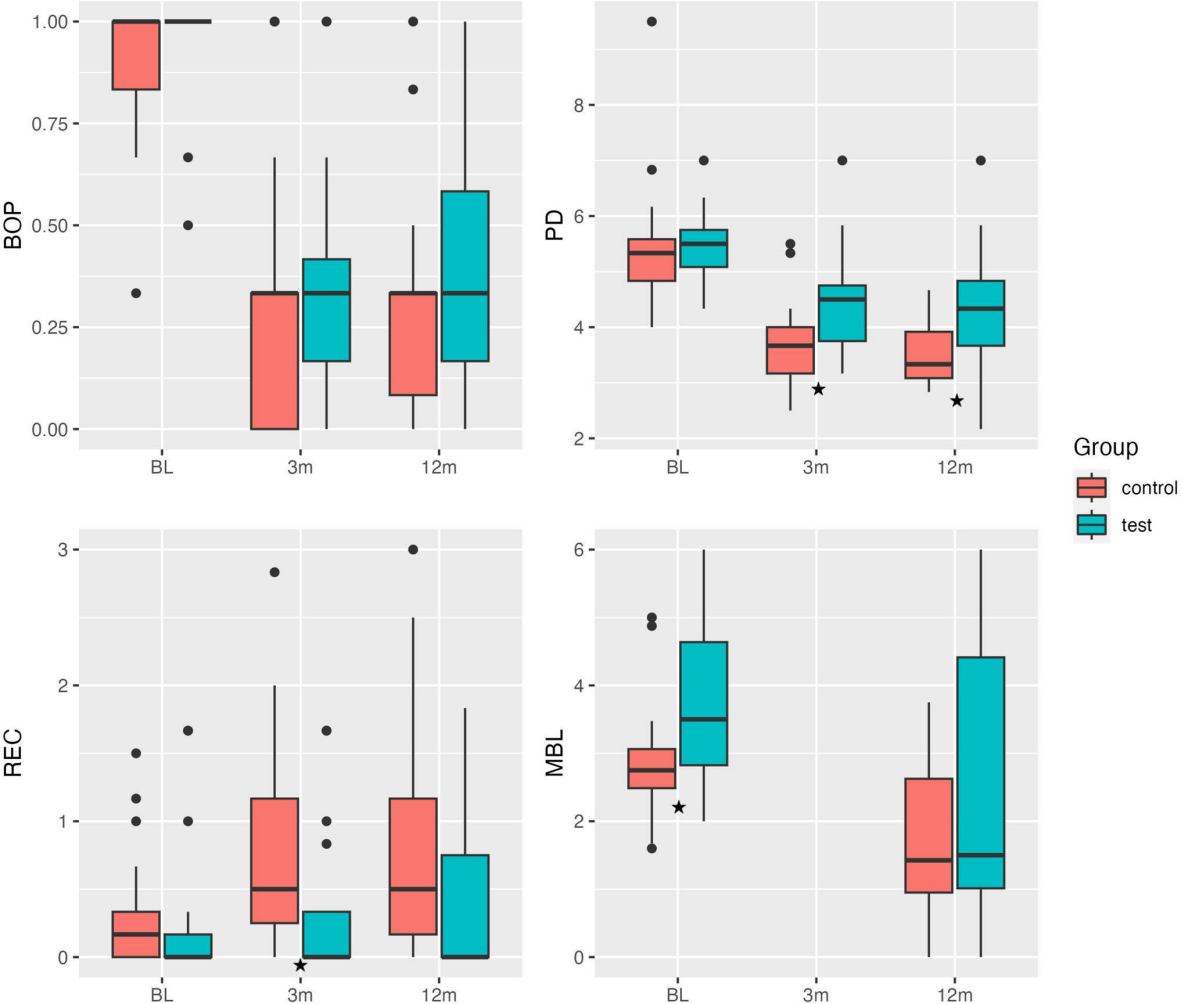
Boxplots showing clinical and radiographic parameters for the test and control groups at Baseline (BL), 3 months (3 m) and 12 months (12 m). Statistically significant differences between the two groups are indicated as “★”

At the 3-month reevaluation visit, both groups showed clinical signs of healing with significant reduction in PD. Yet the control group showed significantly lower PDs; 3.7 mm [3.2, 4.0] compared to the test group at 4.5 mm [3.8, 4.8] (*p* = 0.01). Only 24% of sites presented with BOP, 26.5% from the test group and 21.6% from the control group. Three patients from the test group presented with SUP and had to undergo rescue treatment, while none of the control patients required any additional therapy. Meanwhile, REC values were significantly higher in the control group (0.5 mm [0.3, 1.2]) compared to the test group (0 mm [0.0, 0.3]) (*p* = 0.02). PD values were still significantly lower in the control group at the final reevaluation (3.3 mm [3.1, 3.9] compared to 4.3 mm [3.7, 4.8] for the test group (*p* = 0.01). Marginal bone levels improved in both groups one year post-operatively (Test group 1.5 mm [1.0, 4.4], control group 1.4 mm [1.0, 2.6]) without significant differences between the two groups (Table 2, Fig. 2).

Overall, 22 (57.9%) patients showed complete disease resolution at the end of the observation period. Of these, 14 (36.8%) belonged to the surgical group compared to 8 (21.1%) from the test group, but the difference was not statistically significant (*p* = 0.10). A total of 31.8% of sites presented with BOP, 34.6% from the test group and 29.8% from the control group without significant differences between the groups. Bleeding 5 mm residual pockets were detected in 6 (16.7%) subjects; 3 had received laser therapy while the other 3 had surgical treatment. Three patients (8.3%) presented with at least one residual 6 mm pocket at the 12-month reevaluation visit. Two had received open flap debridement and the third had laser therapy. High plaque scores were recorded for the two control patients with deep residual pockets at the final reevaluation. Regardless, all 3 subjects presented with initial mMBL of 2–2.48 mm and showed radiographic bone gain ranging from 0.5–1.9 mm. A single subject from the test group displayed one residual bleeding pocket of 7 mm at the final visit despite improvement of clinical measurements at the 3-month reevaluation. Nevertheless, mean marginal bone level at this implant showed improvement from 2 mm at baseline to 1.25 mm at 12 months. None of the 32 implants examined at the final reevaluation showed any suppuration nor signs of disease progression.

**Fig. 3:**
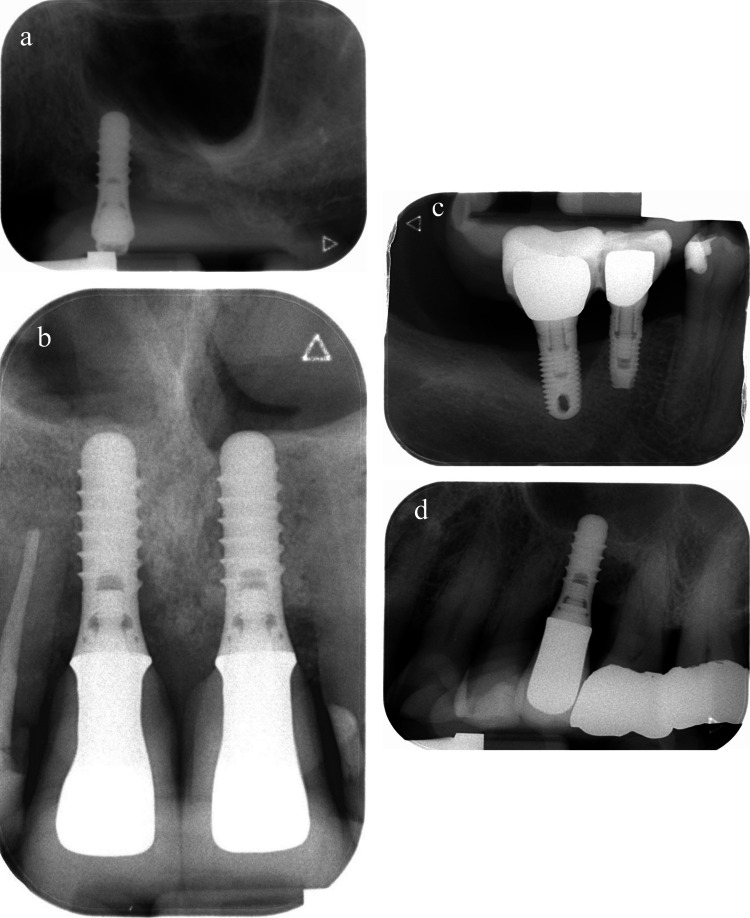
Baseline intra-oral radiographs of the failed test implants. **a**: Subject 11, **b**: Subject 23, **c**: Subject 31, **d**: Subject 35

Laser therapy failed in 4 (23.5%) out of the 17 test participants. One patient (Subject 31) with a hollow screw implant design presented with discomfort and implant mobility during the maintenance phase. Loss of osseointegration was verified both clinically and radiographically, and the implant was removed without complications. The second participant (Subject 11) reported implant loss while removing his RDP 9 months post-operatively. The latter had presented with SUP and a persistent 9 mm pocket at the 3-month reevaluation. Rescue treatment had temporarily succeeded in reducing clinical signs of inflammation and patient’s discomfort. The third participant (Subject 23) also presented with SUP at the 3-month visit. Following three unsuccessful laser rescue attempts, surgical access revealed severe circumferential bone loss and an irrational-to-treat prognosis. The implant was thereby removed with the patient’s consent. The fourth subject (Subject 35) presented with a 9 mm pocket at the 3-month reevaluation visit and opted for surgical treatment with guided bone regeneration due to the severity and progressive nature of the defect. All four failed implants presented with deep pockets (PD 6—9 mm) and advanced radiographic bone loss at baseline (mMBL 3.35—5.5 mm). Table 3 and Fig. 3 show the details of the failed implants. In comparison, none of the control implants showed any signs of failure at the final evaluation, and the difference between the test and control groups in terms of treatment success was statistically significant (*p* = 0.04).

**Table 3:** Details of failed implants in the test group

Subject no	Smoking status	Implant position	Implant type	Loading duration	Prosthetic restoration	Baseline deepest PD	Baseline mMBL	KT > 2 mm	Treatment
11	Yes	25	Solid screw TL	5–10 yrs	RDP	7 mm	5.5 mm	Yes	Explantation due to loss of osseointegration
23	Yes	11	Solid screw TL	3–5 yrs	Screw-retained SC	9 mm	4.38 mm	Yes	Surgical access then explantation
31	No	46	Hollow cylinder	5–10 yrs	Cemented SC	6 mm	3.35 mm	No	Explantation due to loss of osseointegration
35	Yes	25	Solid screw TL	> 10yrs	Cemented SC	9 mm	4.9 mm	No	Surgical access + GBR

*TL* = Tissue-level, *RDP* = Removable dental prosthesis, *SC* = Singe crown, *PD* = Probing depth, *mMBL* = Mean marginal bone loss, *KT* = keratinised tissue, *GBR* = Guided bone regeneration

Twenty-one participants showed bone gain of 1 mm or more at the one year visit; 12 from the control group (63.2%) and 9 (53%) from the test group. None of the participants showed bone loss exceeding 0.5 mm at one year.

### Patient reported outcomes and adverse events

Overall, 18 (47.4%) participants reported at least one adverse event following treatment; 8 (44.4%) test and 10 (55.6%) control subjects, without significant differences between the two groups. However, none had experienced any serious adverse events following either treatment.

Six (35.3%) out of the 17 test patients reported mild transient ache during the day of the first laser treatment. One test subject (5.9%) also reported mild intra-oral swelling on the first day. A single patient (5.9%) reported moderate pain after the first laser treatment and a mild ache 3 days following the second visit. Significant improvement was reported after 1 week with a VAS score of 0 at the third treatment visit. Nevertheless, this subject (#23) presented with SUP during the 3-month visit, and the implant was eventually removed with an irrational-to-treat prognosis, as previously mentioned.

Seven (36.8%) out of 19 control subjects reported gastro-intestinal disturbances attributed to the prescribed antibiotics, and one experienced additional insomnia (5.3%). One patient presented with ulceration following open flap debridement, while another experienced mild post-operative pain and a palatal burning sensation that disappeared a few days after. A single participant presented with flap dehiscence of 0.5–1 mm and slight post-operative discomfort when utilising his RDP 4–7 days following surgical treatment with GBR.

Regarding post-operative discomfort, both groups reported significantly low VAS values. Still, the test group presented with significantly lower VAS scores at the first follow-up compared to the surgical group; IQR 0 [0.0, 0.0] and 3 [0.0, 12.0] respectively (*p* = 0.01). The difference in patients’ overall perception of the treatment, however, was not statistically significant at the one-year visit.

## Discussion

The outcomes of the present study indicated that surgical treatment of peri-implantitis defects is significantly superior to diode laser therapy in terms of treatment success and PD reduction one year post-operatively. Laser treatment failed in 23.5% of cases compared to none in the control group. Meanwhile, complete disease resolution was achieved in 22 (57.9%) patients; 14 (36.8%) belonged to the surgical group compared to 8 (21.1%) from the test group, without statistically significant differences between the two groups.

At the one-year reevaluation visit, all 32 remaining participants presented with significant improvement in both clinical and radiographic parameters. This was in line with the results of Mettraux et al. which showed clinical improvement following diode laser therapy with a significant decrease in PD at both buccal and oral aspects; from 7.5 ± 2.6 mm to 3.6 ± 0.7 mm, and from 7.7 ± 2.1 mm to 3.8 ± 0.9 mm, respectively [47]. Despite reporting continuous fill of the bony defects with corroborating radiographic images, the authors did not present any quantitative data for comparison. Nevertheless, the results of our clinical trial confirms the validity of repeated diode laser therapy as a minimally invasive option for treatment of non-advanced peri-implantitis defects. Other studies have also verified the bactericidal benefits of the diode laser, as well as its ability to maintain titanium’s biocompatibility and surface characteristics [14, 20, 41, 44, 48, 52].

Low-level laser therapy may have contributed to the beneficial clinical outcomes of the current study. The biostimulatory effects of low-level lasers have been discussed in several publications, but their mechanism of action is still unclear. Photobiomodulation may promote wound healing by TGF-β1 signaling and expression of human β-defencin 2 [67]. It can also diminish pain and peri-implant inflammation [1, 4, 15, 45, 47]. Nevertheless, additional clinical trials comparing this protocol with or without adjunctive photobiostimulation are required before reaching any conclusions.

When compared to laser therapy, surgical access initially induced significantly higher mucosal recession. Such outcomes are expected since a regenerative approach could only be employed in 4 (21.1%) out of 19 control cases, while open flap debridement or implantoplasty had been performed in 9 (47.4%) and 6 (31.6%) cases, respectively. A meta-analysis of 26 studies had previously shown that access flaps and resective surgical approaches are significantly associated with higher soft-tissue recession compared to regenerative interventions [62]. Still, the initial differences between the groups diminished below the level of statistical significance at the one-year evaluation.

The authors recognise the limitation of utilising different surgical approaches and the lack of a true control group. This decision was based on the configuration of the peri-implant bony defects, and aimed to provide the best possible outcomes for the patients. GBR was done in the presence of three- or two-wall osseous defects with at least 3 mm infra-bony depth [36, 55, 56]. This was based on a multitude of studies reporting improved clinical and radiographic outcomes when bone substitutes are utilised [18, 36, 37, 59]. Meanwhile, the utilisation of bone grafts renders true marginal bone levels difficult to assess. Therefore, only radiographic bone fill could be evaluated in those cases. A recent meta-analysis has shown improved bone fill and lower mucosal recession when utilising xenogeneic bone grafts as opposed to autogenous ones [55]. On the other hand, a recent randomised controlled clinical trial [32] has reported successful outcomes using only access flaps and systemic antimicrobials. The latter showed comparable results to GBR when treating contained intra-bony defects  [32].

Implantoplasty was performed in cases with supra-crestal bone defects and exposed rough implant surfaces, depending on accessibility and only for non-aesthetic cases. This approach has been proven effective in management of horizontal peri-implant defects [25, 37, 51]. Concerns over titanium particles’ release into the surrounding tissues have been raised, but their clinical implications are still to be determined [49]. The lack of clinical evidence to support a specific treatment protocol, grafting material or implant decontamination method, represents a major obstacle in the development of standardised clinical protocols for management of peri-implantitis.

A randomised controlled clinical trial tested the laser protocol described in the present study in comparison with mechanical debridement alone [61]. Significant improvement in PDs were reported in both groups 6 months post-operatively without additional benefits for the laser therapy. Looking into the details of this trial, the study population included implants with a mean MBL of 2.58 ± 1.03 mm at the deepest site in the test group. In comparison, the present study included implants with a median MBL of 3.5 mm [2.8, 4.6] for the laser group. The higher baseline bone loss in our study probably contributed to such conflicting results. The apparent limitation of laser therapy for treatment of advanced peri-implantitis defects should be taken into account. The additional use of photobiostimulation following implant decontamination could have also contributed to our favorable results. Moreover, Roccuzzo et al. reported a success rate of 41.7% for the laser group and 46.2% for the control group. Treatment success was defined as PD ≤ 5 mm with absence of BOP or PPD ≤ 4 mm without progression of MBL. In comparison, we defined success at a lower cutoff point; PD ≤ 4 mm, complete absence of BOP, SUP, or progressive MBL. This led to an overall rate of 57.9%; with 63.6% belonging to the surgical group compared to 36.4% from the test group.

Other clinical studies have tested different lasers with various wavelengths and power settings for treatment of peri-implantitis. Arisan et al. evaluated diode laser therapy with adjunctive mechanical debridement using plastic curettes in comparison to mechanical debridement alone [6]. The laser was set at 1 W power in a pulsed mode (wavelength 810 nm,energy density 3 J/cm^2^; power density 400 mW/cm2; energy 1.5 J; spot diameter 1 mm; time 1 min). Neither group showed any clinical signs of improvement 6 months after treatment. Moreover, the laser group showed significantly higher MBL at 6 months when compared to the control treatment. This negative effect was attributed to an individual host response and the possible uncontrolled rise in the temperature of the irradiated area, leading to thermal damage and compromised healing [26, 39]. In comparison, we reduced the risk of overheating by continuously moving the fibre tip in a scanning manner as well as using copious saline irrigation during implant irradiation. We also checked the fibre tip every 4–5 s for coagulation. The tip was cut off with scissors when a hot spot was spotted to avoid heat development.

When compared to other lasers, a network meta-analysis [34] showed that diode laser in combination with conventional non-surgical debridement was the treatment most likely effective for reduction of PD compared to Nd:YAG or Er:YAG lasers and non-surgical treatment. Meanwhile, Er:YAG laser in association with mechanical debridement had the highest probability of improving clinical attachment levels and bleeding indexes.

Laser treatment is a non invasive approach generally well accepted by patients with minimal side effects. This was evident in the current study where the test group reported significantly lower post-operative discomfort compared to the surgical group. Nevertheless, both groups showed low VAS values with no significant differences in the overall patient satisfaction at the end of the study period.

**Fig. 4:**
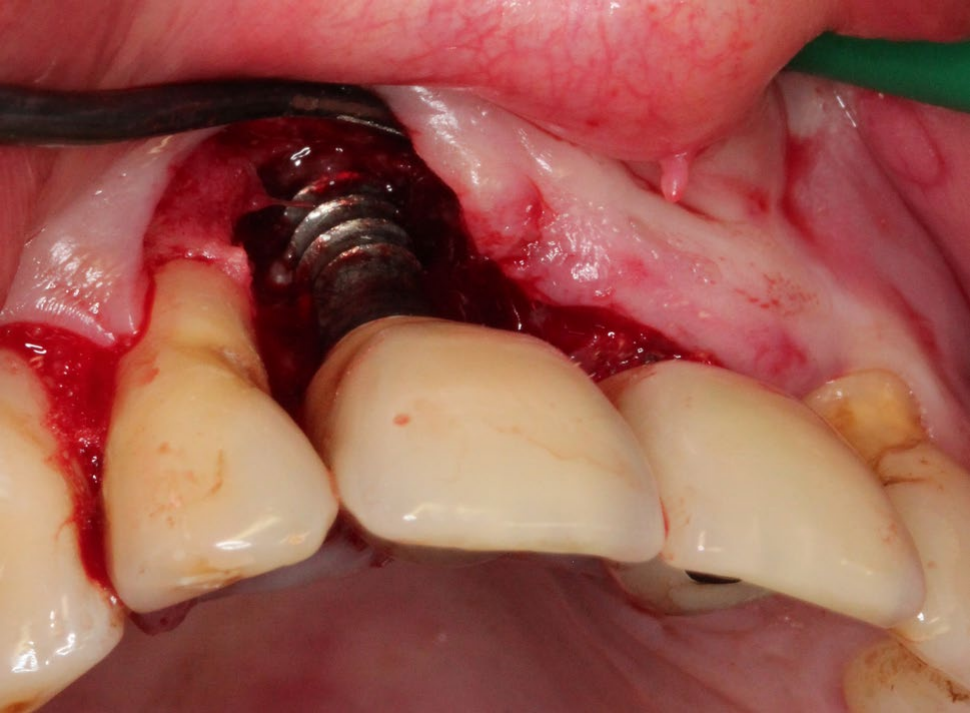
Intra-operative clinical image of subject 23; showing severe circumferential bone loss around implant 11

The results of this study should be interpreted with caution for several reasons. First, the outcomes may have been affected by the limited sample size and the 15% drop-out rate from the test group. Second, initial mMBL was significantly more advanced in the test group, and laser treatment had failed in 4 cases with advanced peri-implantitis defects (Table 3). Nonetheless, neither baseline clinical characteristics nor radiographic measurements could be statistically correlated with the test treatment’s failure. Once again, this could be attributed to the sample size. The accuracy of bone level measurement on intra-oral radiographs and the limitation of two-dimensional imagery should also be taken into account. This was evident in one test case (Subject 23; Table 3) where flap reflection revealed severe palatal bone loss reaching the implant’s apex, and hence a hopeless prognosis (Fig. 4). The defect had been severely underestimated due to the superimposition of the nasal spine and the implant’s body in the intra-oral radiograph (Fig. 3b). A second failure of the laser treatment could be explained by the implant’s hollow-cylindrical design, and the inherent difficulty of disinfecting the contaminated surface (Subject 31, Table 3, Fig. 3c). The latter was the only patient presenting with such implant configuration and, in retrospect, might have been wiser to exclude during the recruitment phase. The remaining failed implants presented with more than 50% mMBL at baseline.

Another factor to consider is the lack of calibration prior to performing clinical measurements. Despite the examiner’s extensive experience, a potential risk of information bias cannot be excluded.

The exclusive prescription of antibiotics for the control group is another important aspect to consider. Several studies have demonstrated the additional beneficial effects of systemic antimicrobials for surgical treatment’s success, irrespective of the implants’ decontamination protocol [10], [53]. This probably contributed to the control treatment’s significant clinical improvement and high success rate. However, the routine use of antibiotics has been reconsidered in recent publications due to the growing concerns over bacterial resistance [11, 53, 68, 70], and in retrospect, might have been better avoided. On the other hand, the laser treatment protocol was based on a case series [47] which showed significant clinical improvement 2 years following non-surgical treatment of peri-implantitis with repeated diode laser application. The authors wished to test the exact treatment method without additional prescription of systemic antibiotics. Nevertheless, we recognise the limitation this confounding variable represents to our study.

In conclusion, surgical treatment of peri-implantitis demonstrated significant benefits over laser therapy in terms of success rate and PD reduction. Complete disease resolution was higher following surgical treatment, but the difference was not statistically significant. Nevertheless, diode laser therapy, as described in this study, could represent a non-invasive alternative for treatment of non-advanced peri-implantitis defects. Larger scale randomised controlled clinical trials are still required to evaluate the limits of diode laser therapy and the prognostic factors affecting its long-term success.

## Data Availability

No datasets were generated or analysed during the current study.
